# Synthesis*, ex Vivo* and *in Vitro* Hydrolysis Study of an Indoline Derivative Designed as an Anti-Inflammatory with Reduced Gastric Ulceration Properties

**DOI:** 10.3390/molecules14093187

**Published:** 2009-08-26

**Authors:** Man Chin Chung, Jean Leandro dos Santos, Ednir Vizioli Oliveira, Lorena Blau, Renato Farina Menegon, Rosângela Gonçalves Peccinini

**Affiliations:** 1 Lapdesf - Laboratório de Pesquisa e Desenvolvimento de Fármacos, Departamento de Fármacos e Medicamentos, Faculdade de Ciências Farmacêuticas – UNESP Rodovia Araraquara Jaú Km. 01, 14801-902, Araraquara, SP, Brazil; E-mail: jeanleandrosantos@yahoo.com.br (J.L.d.S.); 2 Departamento de Princípios Ativos, Naturais e Toxicologia, Faculdade de Ciências Farmacêuticas – UNESP Rodovia Araraquara Jaú Km. 01, 14801-902, Araraquara, SP, Brazil; E-mail: peccinin@fcfar.unesp.br (R.G.P.)

**Keywords:** indolinone, pro-drug, anti-inflammatory, hydrolysis, diclofenac

## Abstract

The compound 1-(2,6-dichlorophenyl)indolin-2-one (**1**), planned as a pro-drug of diclofenac (**2**), was easily synthesized in 94% yield by an intramolecular reaction in the presence of coupling agent (*i.e.,* EDC). Compound **1** showed anti-inflammatory and analgesic activity without gastro-ulcerogenic effects. The chemical and enzymatic hydrolysis profile of the lactam derivative **1** does not indicate conversion to diclofenac (**2**). This compound is a new non-ulcerogenic prototype for treatment of chronic inflammatory diseases.

## 1. Introduction

Non-steroidal anti-inflammatory drugs (NSAIDs) are widely used in the treatment of inflammation, pain and arthritis. The beneficial effect is associated with inhibition of cyclooxygenases (COX) that convert arachidonic acid into prostaglandins in inflammatory processes [1]. The major limitation of long-term therapeutic use of NSAIDs (COX-1 inhibitors) is their gastrotoxicity. This side effect produced by NSAIDs are believed to involved two different mechanism: inhibition of prostaglandin synthesis in the stomach, responsible for inducing mucus production and a local action exerted by direct contact of the drugs with the gastric mucosa due the acidic nature of the NSAIDs [2].

The use of latentiation as a molecular modification strategy provides NSAID produgs with improved safety profiles [3,4]. The prodrug approach afforded compounds with better anti-inflammatory activity, differentiated pharmacokinetic profiles and reduced gastric ulcerogenic activity [5,6,7,8,9]. Using the prodrug approach, one strategy that could be useful is to temporarily mask the carboxylic acid function of the NSAIDs so the prodrug hydrolyzes *in vivo* to release the active parent NSAID [10,11,12].

**Scheme 1:**
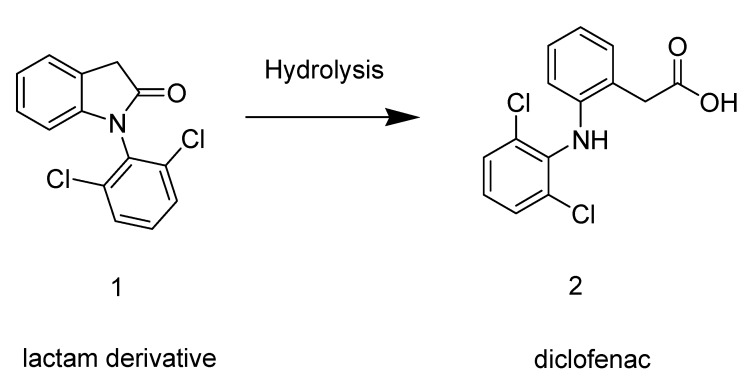
Prodrug hydrolysis leading to the parent drug diclofenac.

In this context, we have conceived a new indolinonic compound **1** that after hydrolysis could regenerate the parent drug diclofenac (**2**) (Scheme 1). The carboxylic acid function of diclofenac was thus masked and the new molecule **1** was expected to have anti-inflammatory activity with less gastro-ulcerogenic effect. The anti-inflammatory and analgesic activities were evaluated using classical models. In addition, we have studied the *in vitro* and *ex vivo* hydrolysis profile of the compound **1**.

## 2. Results and Discussion

### 2.1. Synthesis

The compound were prepared through an intramolecular reaction of diclofenac using 1-ethyl-3-[3-dimethylaminopropyl]carbodiimide hydrochloride (EDC) as coupling reagent, in room temperature for 30 min. After, the reaction mixture was diluted with dichloromethane and washed with distilled water. The solvent removed by evaporation resulting in a 94% yield of 1-(2,6-dichlorophenyl)indolin-2-one (**1**). The purity of synthesized compound was checked by thin layer chromatography (TLC) and elemental analyses. The structure was characterized by nuclear resonance magnetic (NMR), infrared spectroscopy (IR) and mass spectrometry. The elemental analysis results were within ±0.4% of the theoretical values. The ^1^H-NMR spectrum showed the methylene protons at δ 3.87 (s, 2H), while the aromatic protons appeared at δ 6.38-7.74. The IR spectrum of compound **1** showed the presence of a lactam carbonyl at 1,732 cm^-1^. The C-N and C-Cl stretching appeared at 1,612 cm^-1^ and 783 cm^-1^, respectively.

### 2.2. Hydrolysis

The chemical and enzymatic hydrolysis of the synthesized lactam was carried out in aqueous buffer solutions (pH 1.2 and 7.4) and in human serum (80%), respectively. A HPLC (UV-Vis detector) method, specific for the estimation of the released parent drug diclofenac (**2**) and lactam derivative **1** was developed.

**Figure 1:**
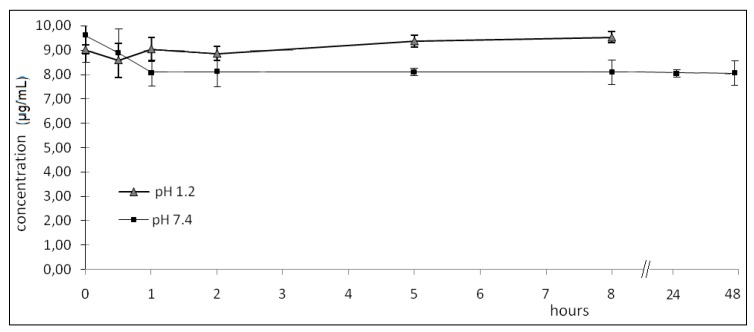
Hydrolysed of lactam derivatives (µg/mL) in buffer pH 1.2 and 7.4 (data are represented as mean ±S.E.M., *n* = 6, *P* < 0.05).

In pH 1.2 buffer the lactam derivative was found intact after 8 h, with no observable hydrolysis. Based on these observations, it was concluded that the stomach would not be exposed to the free carboxylic group of the NSAID (diclofenac) since the lactam would not be cleaved in the stomach (Figure 1).

In pH 7.4 buffer the lactam derivative did not observably hydrolyze after 48 h. The compound could thus be absorbed in its intact form without biotransformation to diclofenac in the intestine. Furthermore, these data indicate that the lactam is stable in plasmatic pH after absorption for at least 48 h. It was concluded from these observations that these derivatives survived the GI pH conditions successfully.

**Figure 2:**
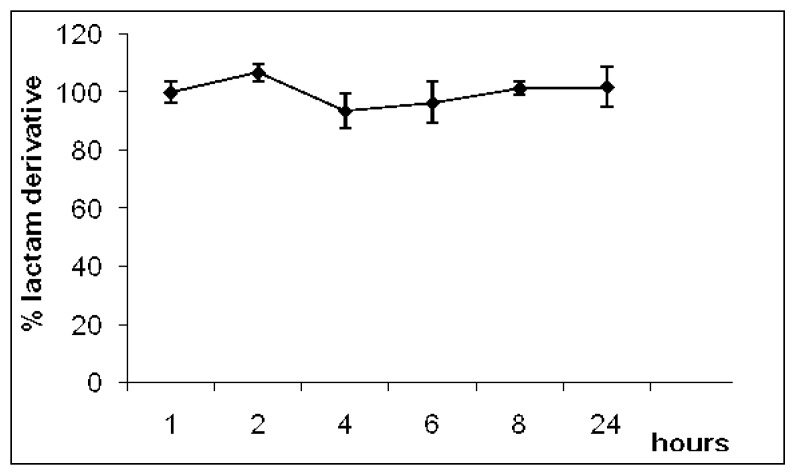
Hydrolysed percentage of lactam derivative in plasma (data are represented as mean ±S.E.M., *n* = 8, *P* < 0.01).

The plasma hydrolysis study was carried out to evaluate the influence of enzymatic hydrolysis in plasma during 24 h. Enzymes such as amidases have been shown to hydrolyze lactam rings. However, no hydrolysis was observed in plasma (pH 7.4; 37 °C), indicating that the lactam derivative did not undergo enzymatic biotransformation in plasma (Figure 2).

**Figure 3:**
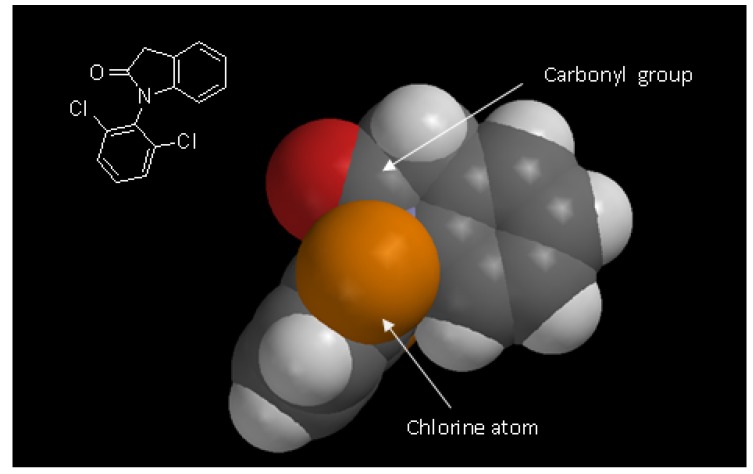
Space filling model showing the steric hindrance of carbonyl caused by chlorine atom to nucleophilic attack in hydrolysis reaction (gray: carbon; red: oxygen; white: hydrogen; orange: chlorine structure obtained by Spartan Pro PC 1.0.5 program – wavefunction, inc.).

The absence of lactam hydrolysis could be due to a combination of electronic and steric factors. Hydrolyses of amides and lactams are slower when compared to ester or lactone hydrolyses because the oxygen atom attached to the carbonyl (in esters and lactones) is more electron withdrawing that the nitrogen atom in amides. So the ester carbonyl group becomes more deficient electronically and suffers more easily nucleophilic attacks in hydrolysis reactions than the respective amides. This could explain, electronically, the greater stability of amides and lactams. On the other hand, structurally the carbonyl of this lactam is more sterically blocked, decreasing the chemical hydrolyses of the substrate **1**. The presence of two chlorine atoms attached to the aromatic ring blocks the access of nucleophiles to the carbonyl of the lactam to hydrolysis reactions (Figure 3).

This factors confers stability and could explain why the compound 1-(2,6-dichlorophenyl)indolin-2-one (**1**) remains unaffected by chemical hydrolysis in the various pH conditions of biological fluids.

### 2.3. Pharmacological evaluation

**Figure 4:**
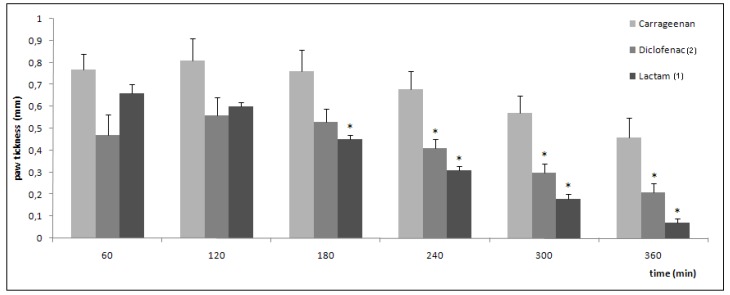
Anti-inflammatory activity in carrageenan-induced paw edema in rats (data are represented as mean ±S.E.M., *n* = 6, *P < 0.05 with respect to carrageenan; dose of compounds = 100 μmol/kg).

Inhibition of swelling in carrageenan-induced edema in the rat paw by oral administration of the drugs is shown in Figure 4. After 3 hours, the anti-inflammatory activity of lactam **1** was statistically significant when compared with the carrageenan group. After 4 hours, the anti-inflammatory activity of both compounds is significant. It has been noted that the anti-inflammatory activity of lactam **1** and diclofenac (**2**) is comparable. All results were statistically significant when compared to aqueous solution of sodium carboxymethylcellulose (0.5% w/v) used as control (not shown).

The anti-nociceptive activities of diclofenac (**2**) and its lactam derivative **1** were measured by their ability to inhibit acetic acid-induced writhing in mice. The vehicle control was considered to exhibit 100% writhing, and the protection afforded by the parent drug and its derivative was calculated on a percentage basis. The percentage of protection was calculated using Equation (1):

Protection (%) = 100 – [number of writhings in test / number of writhings in control × 100] (1)

According to Table 1, the lactam **1** possesses analgesic protection equivalent to 48.1% of control values, which was less than diclofenac (65.2%) in the model used. These results point to the fact that lactam **1** seems to be more effective as an anti-inflammatory than as an analgesic compound when compared to the parent drug (diclofenac).

The ulcerogenicity assay was performed using rats administered 100 μmol/kg^–1^ of each drug. It was possible to observe a smaller number of ulcers in animals treated with lactam **1**, compared to the animals treated with diclofenac (Table 2).

**Figure 5:**
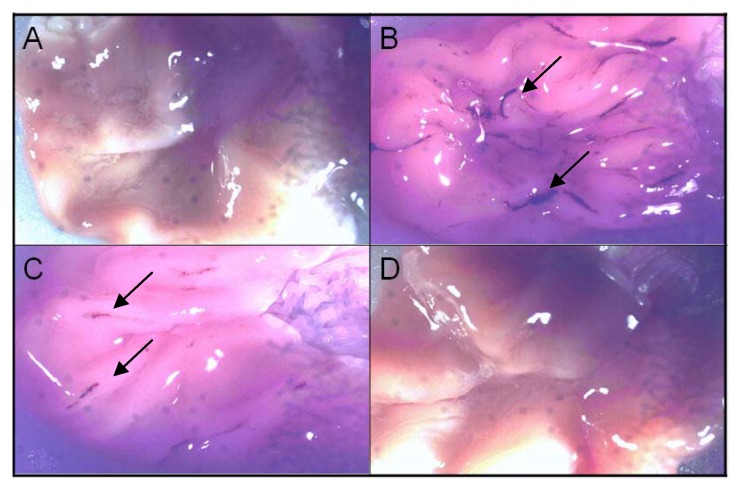
Stomach ulcerogenicity of compounds at 100 μmol kg^–1^ in rats. A) DMSO control; B) diclofenac (score 4; lesions > 2 mm pointed by arrows); C) celecoxib (score 2; lesions between 1–2 mm pointed by arrows) and; D) lactam derivative (**1**) absence of ulceration.

Celecoxib was used as a COX-2 inhibitor at the same concentration. Animals treated with diclofenac developed an average of 69 ulcerogenic lesions with only one administration and around 6.7% of the lesions were considered to be large (higher than 2 mm in diameter). This allowed to the classification of lesions, which was scored depending on the severity of mucosal damage. Celecoxib administration, at the above concentration, led to the development of around six lesions –50% of which were puntiform (lesions <1mm) and 50% of small lesions (1.0-2.0 mm). Celecoxib was classified in this assay as scoring a 2 for lesion development. The lactam derivative did not cause any mucosal damage and was classified with a score of 0 (Figure 5). The results were obtained with an average of six animals analyzed per group. These findings suggest that masking of the carboxylic function of the diclofenac successfully decreased gastro-ulcerogenicity.

**Table 1:** Percentage protection in acetic acid induced writhings by diclofenac and lactam **1** in mice (dose: 100 μmol kg^–1^).

Treatment	Number of writhings (average)	% protection
control	62 ± 1.9	-
diclofenac	21.6 ± 0.8^≠^	65.2
lactam **1**	29.7 ± 0.5^≠^*	48.1

^≠^ P<0.01, compared with control; * P<0.05, compared with diclofenac.

**Table 2:** Ulcerogenic effect of diclofenac, celecoxib and lactam (**1**) in rats (n = 6, mean ±S.D.).

compound	number of ulcers	< 1 mm	1-2 mm	>2 mm
diclofenac	69 ± 6.15	62 ± 7.5 (89%)	2.9 ± 2.5 (4.3%)	4.6 ± 1.9 (6.7%)
celecoxib	6 ± 1.1^*^	3 ± 1.5 (50%)	3 ± 0.9 (50%)	-
lactam **1**	0^*^	-	-	-

* Significant difference compared to group that received diclofenac. P < 0.05 (Tukey’s test).

## 3. Experimental

### 3.1. General

Melting points were taken with an Electrothermal melting point apparatus (SMP3 Bibby Stuart Scientific) in open capillary tubes and are presented as uncorrected values. Infrared spectra (KBr discs) were obtained on a FTIR-8300 Shimadzu, and frequencies were expressed in cm^-1^. ^1^H-NMR spectra were scanned on a Bruker DRX-400 (400 MHz) NMR spectrometer using DMSO-d_6_ as solvent. Chemical shifts were expressed in ppm (parts per million) relative to tetramethylsilane. Elemental analyses (C, H and N) were performed on a Perkin Elmer model 240C analyzer, and the data were within ±0.4% of the theoretical values.

### 3.2. Materials

A diclofenac standard was purchased from the EMS-Sigma Pharma (Hortolândia, SP, Brazil). *N*-(3-dimethylaminopropyl)-*N*-ethylcarbodiimide (EDC) (Sigma-Aldrich, St. Louis, MO, USA), potassium phosphate and trichloroacetic acid (TCA) were purchased from Labsynth (São Paulo, SP, Brazil). Acetonitrile was obtained from J.T. Baker (Phillipsburg, NJ, USA).

### 3.3. Animals

Male Wistar rats (200-250 g) and Swiss albino mice (25–30 g) were housed at a constant temperature (23 ± 1.8 ºC), humidity (55 ± 5%) and a light cycle (12/12 h) with food and water *ad libitum*. Experiments were conducted during the light phase. The study protocol was approved by The Research Ethics Committee of the School of Pharmaceutical Science, UNESP, Araraquara (Process 27/2006).

### 3.4. Synthesis of 1-(2,6-dichlorophenyl)indolin-2-one *(1)*

Diclofenac (**2**, 315 mg, 1.1 mmol) and 1-ethyl-3-[3-dimethylaminopropyl]carbodiimide hydrochloride (EDC, 226 mg, 1.2 mmol) were placed in dichloromethanea (20 mL) and the reaction mixture was stirred for 30 min at room temperature. Then, the reaction mixture was diluted with dichloromethane (50 mL) and washed with distilled water (3 x 20 mL). The organic phase was dried with sodium sulfate and the solvent removed by evaporation resulting in 278 mg (94% yield) of 1- (2,6-dichlorophenyl)indolin-2-one as a red substance; m.p.: 121 - 123 °C; ^1^H-NMR: δ 3,87 (s, 2H), 6,38 (d, 1H, *J*=7.63 Hz), 7,08 (dt, 1H, *J*=7.67 Hz and 1.05 Hz), 7,20 (dt, 1H, *J*=7.67 Hz and 1.05 Hz), 7,38 (d, 1H, *J*=7.67 Hz), 7,74 (d, 2H, *J*=8.13 Hz), 7,60 (dd, 1H, *J*=8.13 Hz); IR): 1,732 (C=O lactam), 1,612 (CN), 783 and 750 (C-Cl) cm^-1^; MS-EI: 278 (m/z). Calculated for C_14_H_9_Cl_2_NO: C, 60,46; H, 3,26; N,5,04. Found: C, 60,2; H, 3,21; N, 5,2.)

### 3.5. In vitro hydrolyses of 1-(2,6-dichlorophenyl)indolin-2-one *(1)* in buffer and human plasma

#### 3.5.1. Analytical protocol

The concentrations of 1-(2,6-dichlorophenyl)indolin-2-one (**1**) and diclofenac (**2**) in human plasma and buffer were measured by a HPLC method. The HPLC system used was a Shimadzu model LC-10AD equipped with a model SPD-10A UV-Vis detector (Shimadzu). The compounds were separated on a reverse phase C18 column (5 μm particle, 250 mm × 4.6 mm I.D) Shimadzu Shim-pack CLC-ODS (M) by running an isocratic flow of 65% acetonitrile and 35% 25 mM sodium acetate/acetic acid aqueous buffer solution at pH 4.0, with a flow rate of 1.5 mL/min over 6 min and detection at 280 nm. The calibration curve was linear (r^2^ = 0.9999; n = 8) in the range 0.1–20 µg/mL.

#### 3.5.2. 1-(2,6-dichlorophenyl)indolin-2-one (**1**) *in vitro* hydrolyses in buffer and human plasma

For buffer hydrolysis, an appropriate amount of pure 1-(2,6-dichlorophenyl)indolin-2-one (**1**) was weighed and diluted in sodium acetate (pH 1.2 and 7.4) to a concentration of 10 μg/mL. The samples of plasma, spiked with stock acetonitrile solution of 1-(2,6-dichlorophenyl)indolin-2-one (**1**), were prepared at concentration of 10 μg/mL and subjected to a constant agitation in a shaker at 37 ºC during the entire assay. Briefly for the HPLC analyses, the samples were centrifuged at 2,800 rpm for 10 min (room temperature). To the supernatant, NaOH (pH 10), NaCl and ethyl acetate (4 mL) were added, vortexed for 2 min and centrifuged at 3,000 rpm for 10 min. The organic phases were evaporated to dryness under a flow of nitrogen at room temperature. The residues obtained were resuspended in 300 µL acetonitrile and injected into the HPLC system as following:

pH 1.2: zero; 0.50; 1.00; 2.00; 5.00; 8.00 h (08 h, considered to be the maximum time for this drug to be in gastrointestinal tract).
pH 7.4: zero; 0.50; 1.00; 2.00; 5.00; 8.00; 24.0; 48.0 h.
Plasma: zero; 0.25; 0.50; 1.00; 2.00; 4.00; 8.00; 24.0 h.

All samples were done in triplicate and the results are expressed by the average of the concentrations of the solutions.

#### 3.5.3. Statistical analysis

The data were expressed as mean ±SEM, and analyzed by one-way analysis of variance (ANOVA) followed by Tukey’s test for multiple comparisons among groups (Sigma-Stat software). The calibration and variation coefficient (CV%) curve calculations were performed using the Origin® program.

### 3.6. Anti-inflammatory activity

The anti-inflammatory activity was evaluated using carrageenan-induced rat paw edema method [13]. Wistar rats (150–200 g) were divided into three groups of six animals each. Group I served as a control group without using any drug, group II received diclofenac (**2**) at 100 μmol kg^–1^, and group III received lactam **1** at 100 μmol kg^–1^ as a homogeneous suspension in an aqueous solution of sodium carboxymethylcellulose (0.5% w/v), where the dose was molecularly equivalent to diclofenac. Each animal received 0.75–1.0 mL orally of the respective drugs.Thirty minutes after the administration of drugs, each rat received a subplantar injection of 0.1 mL of 1% carrageenan solution in its left hind paw. The measurement of the hind paw volume was carried out using a plethysmometer before any treatment (*V*_o_) and in at any interval (*V*_t_) after the administration of drugs. All the results are expressed as mean ±S.E.M. Statistical analysis was performed with ANOVA followed by Tukey’s test.

### 3.7. Analgesic activity

Analgesic activity was evaluated using acetic acid to induce writhing [14] in Swiss albino mice (25–30 g) of either sex. A 1% v/v solution of acetic acid was used as a writhing inducing agent. Test compounds were administered orally 1 h prior to acetic acid injections. The number of writhings for 30 min duration in control and test compounds was counted and compared. Analgesic activity was measured as a percent decrease in writhings in comparison to controls. Mice were divided into three groups of six animals each. Group I served as a control group without any drug, while group II received diclofenac (100 μmol kg^–1^) and group III received lactam at (100 μmol kg^–1^).

Each animal received 0.3–0.4 mL orally of the respective drugs prepared as a homogeneous suspension in aqueous solution of sodium carboxymethylcellulose (0.5% w/v). Acetic acid was administered intraperitoneally at a dose of 1 mL/100 g body weight of the animal. All the results are expressed as mean ±S.E.M. Statistical analysis was performed with ANOVA followed by Tukey’s test.

### 3.8. Ulcerogenicity

Gastrointestinal toxicity was determined using the method as described by Cioli *et al*. [15]. The studies were carried out on healthy Wistar rats (150–200 g) at 100 μmol kg^–1^. The animals were divided into four groups of six animals each, group I served as a control and received vehicle only. Group II received pure diclofenac at 100 μmol kg^–1^. Group III received lactam at 100 μmol kg^–1^. Group IV received celecoxib at 100 μmol kg^–1^. The animals were fasted 8 h prior to a single dose of either the control or test compounds, given free access to food and water and sacrificed 17 h later. The gastric mucosa of the rats was examined using a 4× binocular magnifier. The lesions were counted and divided into large (greater than 2 mm in diameter), small (1–2 mm) and puntiform (less than 1 mm). For each stomach the severity of mucosal damage was assessed according to the following scoring system: 0- no lesions or up to five puntiform lesions; 1- more than five puntiform lesions; 2- one to five small ulcers; 3- more than five small ulcers or one large ulcer; 4- more than one large ulcer. The mean score of each treated group minus the mean score of the control group was considered as the ‘severity index’ of gastric damage. Statistical analysis was performed with ANOVA followed by Tukey’s test.

## 4. Conclusions

The lactam derivative 1-(2,6-dichlorophenyl)indolin-2-one (**1**) was designed to have anti-inflammatory and analgesic activity comparable to the diclofenac, with reduced gastrotoxicity effects. The advantage of this compound is its absence of ulcerogenicity, which could be due to the masking of the carboxylic acid function in a lactam moiety. The treatment of chronic inflammatory process demands NSAIDs utilization, and frequently the gastro-ulceration is a common side effect of these drugs. Therefore, the discovery of new compounds with anti-inflammatory and analgesic activities without gastrotoxicity is important for the treatment of chronic inflammatory diseases such as arthritis.

*In vitro* and *ex vivo* (plasma) hydrolysis demonstrated that lactam derivatives are stable and are not metabolized to diclofenac. The next step is the study of pharmacokinetic profile of lactam with the aim to investigate *in vivo* hydrolyses.
